# CT-Guided Percutaneous Step-by-Step Radiofrequency Ablation for the Treatment of Carcinoma in the Caudate Lobe

**DOI:** 10.1097/MD.0000000000001594

**Published:** 2015-10-02

**Authors:** Jun Dong, Wang Li, Qi Zeng, Sheng Li, Xiao Gong, Lujun Shen, Siyue Mao, Annan Dong, Peihong Wu

**Affiliations:** From the Department of Medical Imaging & Image Guided Therapy, Sun Yat-Sen University Cancer Center, State Key Laboratory of Oncology in South China, Collaborative Innovation Center for Cancer Medicine, East Dong Feng Road 651, Guangzhou, Guangdong 510060, PR China (JD, WL, QZ, SL, LS, SM, AD, PW); and Department of Medical Statistics and Epidemiology School of Public Health, Sun Yat-sen University, Zhongshan 2nd Road, 74, Guangzhou, Guangdong 510080, PR China (XG).

## Abstract

The location of the caudate lobe and its complex anatomy make caudate lobectomy and radiofrequency ablation (RFA) under ultrasound guidance technically challenging. The objective of the exploratory study was to introduce a novel modality of treatment of lesions in caudate lobe and discuss all details with our experiences to make this novel treatment modality repeatable and educational.

The study enrolled 39 patients with liver caudate lobe tumor first diagnosed by computerized tomography (CT) or magnetic resonance imaging (MRI). After consultation of multi-disciplinary team, 7 patients with hepatic caudate lobe lesions were enrolled and accepted CT-guided percutaneous step-by-step RFA treatment.

A total of 8 caudate lobe lesions of the 7 patients were treated by RFA in 6 cases and RFA combined with percutaneous ethanol injection (PEI) in 1 case. Median tumor diameter was 29 mm (range, 18–69 mm). A right approach was selected for 6 patients and a dorsal approach for 1 patient. Median operative time was 64 min (range, 59–102 min). Median blood loss was 10 mL (range, 8-16 mL) and mainly due to puncture injury. Median hospitalization time was 4 days (range, 2–5 days). All lesions were completely ablated (8/8; 100%) and no recurrence at the site of previous RFA was observed during median 8 months follow-up (range 3–11 months). No major or life-threatening complications or deaths occurred.

In conclusion, percutaneous step-by-step RFA under CT guidance is a novel and effective minimally invasive therapy for hepatic caudate lobe lesions with well repeatability.

## INTRODUCTION

The caudate lobe of the liver located deeply in the central portion of the back, belongs to the Couinard I segment, and is sandwiched in a “valley of blood” among the first, second, and third portal.^1^ The caudate lobe contains a unique Glisson system and unique venous system different from other liver lobes.^2^ Primary hepatic tumors or metastases located in the caudate lobe are not infrequent,^3^ and surgical resection is one of the most effective methods of treating tumors in the caudate lobe. However, the location of the caudate lobe and its complex anatomy make caudate lobectomy technically challenging for many surgeons^4^; such surgery also requires a lengthy operation and has high risks of bleeding and postoperative recurrence.^5^ With the continuous development of medical technology in recent years, the radical treatment radiofrequency ablation (RFA) has emerged as a novel method for treating caudate lobe tumors. Due to the risk of damaging neighboring large blood vessels during puncture, RFA is usually guided under ultrasound or combined with laparoscopy.^1,6^ Though ultrasound can guide the puncture in real time, it cannot clearly and accurately indicate the ablated area lacking well vision, which increases the recurrence rate for caudate lobe tumors after ablation. An urgent better way was required. CT provides high-quality images, which can accurately indicate the target tumor position and map the ablated zone. CT-guided RFA has been performed on tumors in other sites of the liver for many years.^7^ However, to the best of our knowledge, there are no detailed and believable reports on CT-guided percutaneous step-by-step RFA for caudate lobe tumors.

In the exploratory study, we performed percutaneous RFA under CT guidance in patients with hepatic caudate lobe tumors and clearly described the whole procedure and discussed feasibility, difficulties, dangers, and complication we might meet in the procedure with our experiences.

## MATERIAL AND METHODS

### Study Population

This research met the basic standards of the Declaration of Helsinki and was approved by the Institutional Review Board of Sun Yat-sen University Cancer Center. All patients clearly understood the potential risks and voluntarily signed the informed consent. The methods were carried out in accordance with the approved guidelines.

**FIGURE 1:**
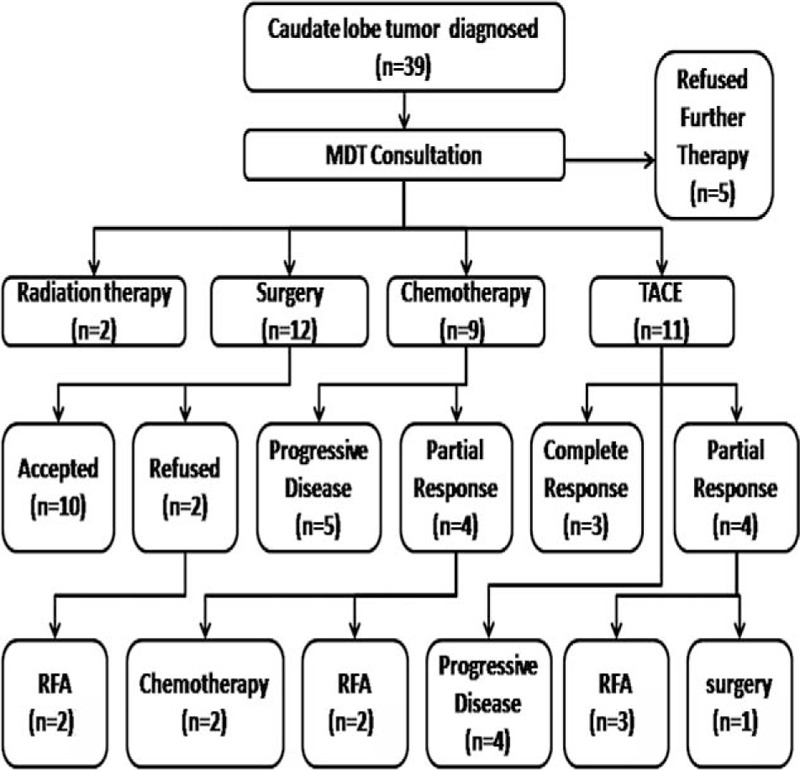
Treatment flow chart of 39 patients with hepatic lesions in caudate lobe. It clearly showed the treatment patients received and the number of patients who received certain treatment. Patients received RFA finally were finally enrolled into the study. RFA = radiofrequency ablation.

Between February 1, 2010, and December 20, 2012, a total of 39 patients were diagnosed with hepatic caudate lobe tumors in our hospital. The clinicopathological features of the patients were summarized in Table 1. Of these 39 patients, 9 cases with hepatic carcinoma had suffered recurrence after transcatheter arterial chemoembolization (TACE), 6 cases had suffered recurrence after TACE and surgery, 10 cases had suffered recurrence after surgical resection, 9 cases were metastases from other carcinomas, and 5 were newly diagnosed. After consultation of the multidisciplinary team, 12 patients were suggested with surgical resection (10 accepted, 2 refused and underwent RFA directly), 9 patients with liver metastases accepted chemotherapy, 2 patients with portal vein tumor thrombi underwent radiotherapy, 11 patients with unresectable tumors underwent TACE, and 5 patients had refused further treatment. Subsequently, 7 patients accepted RFA treatment of the tumors in the caudate lobe (3 cases after TACE, 2 cases after chemotherapy, and 2 cases directly underwent RFA). Further details are shown in Figure 1.

**TABLE 1:**
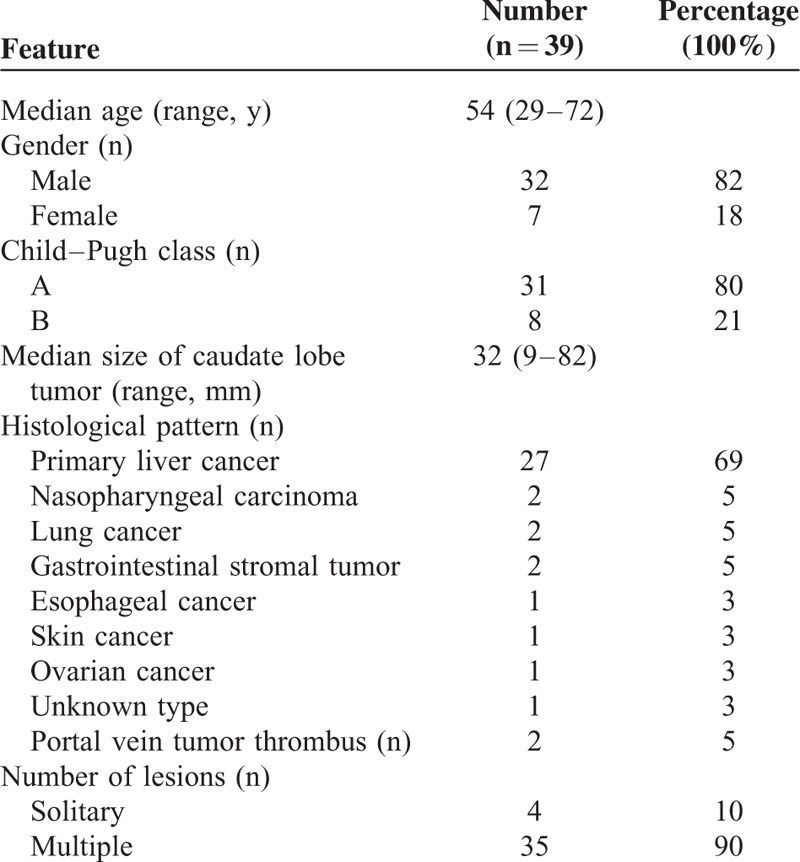
Clinicopathological Features of the Patients With Caudate Lobe Tumors

### Diagnosis

Regardless of whether the hepatic lesions were primary or secondary tumors, the patients were initially diagnosed using enhanced-contrast computed tomography (CT), magnetic resonance imaging (MRI), or both according to standard CT and MRI criteria. Fine needle cytology (FNC) was considered if the imaging appeared atypical and the differential diagnosis was unspecific.

For primary liver cancer, family history, clinical symptoms, physical examination, and serum α-fetoprotein (AFP) levels were also considered to assist the diagnosis. Elevated AFP levels (> 400 ng/mL) were considered indicative of liver cirrhosis in the absence of FNC. For liver metastases, histological diagnosis of the primary tumor, typical imaging protocols, and assessment of serum tumor biomarkers were used to assist the diagnosis of liver metastases.

### Enrollment Criteria

Patients who were suitable candidates for RFA: (1) no extrahepaticinvasion or no invasion to adjacent normal tissues (capsule, bile duct, portal vein, inferior vena cava), (2) safety margin > 5 mm if lesions located near to capsule, bile duct, portal vein, inferior vena cava (no matter the size), (3) East Coast Oncology Group (ECOG) performance status value ≤ 2, (4) Child–Pugh grade ≤ B, (5) who refused other further therapies and voluntarily accepted RFA treatment after being informed of the potential risks.

### Patient Preparation

Before RFA, a series of panel including a complete blood count, liver function tests, renal function tests, and assessment of serum tumor biomarkers and prothrombin time were performed. Digestive secretion was inhibited 12 h before surgery by pantoprazole to reduce the risk of complications if there was ulcer disease. Food and water were withheld in the 12 h before the procedure to reduce the risk of complications associated with intravenous anesthetics.

### RFA Approaches

**FIGURE 2:**
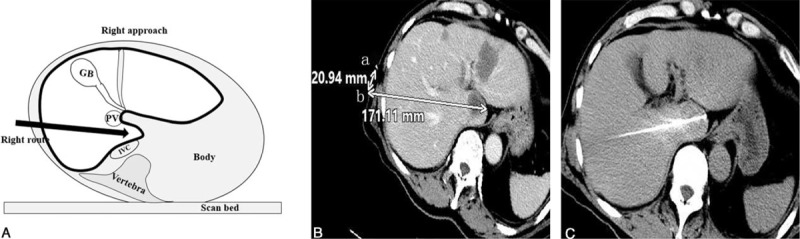
Illustration of the right approach for puncture. Figure A showed the posture for patients on the CT scan bed and the planned right approach for RFA probe to the lesion. Figure B showed the designed puncture route on the preoperative contra-enhanced CT image (white arrow in liver). Once puncture site (b) was set, the distance (20.94 mm) from the imaging marker (a) was determined. The appropriate angle and the depth for puncture (171.11 mm) were designed to guide the operator to perform accurate puncture and to avoid puncture injury to the vessels. Figure C showed the position of the RFA probe after puncture in liver. CT = computerized tomography, RFA = radiofrequency ablation.

In contrast to ultrasound-guided RFA, the general approaches included a right or dorsal approach. The right approach was carried out via an intercostal right lateral puncture with the patient in a left lateral position. The transhepatic tract passed through the right liver and narrow portacaval gap to reach the caudate lobe tumor without passing through vessels or the biliary system (Fig. 2). This approach required special care and accurate planning to avoid puncturing the major vessels in the liver.

**FIGURE 3:**
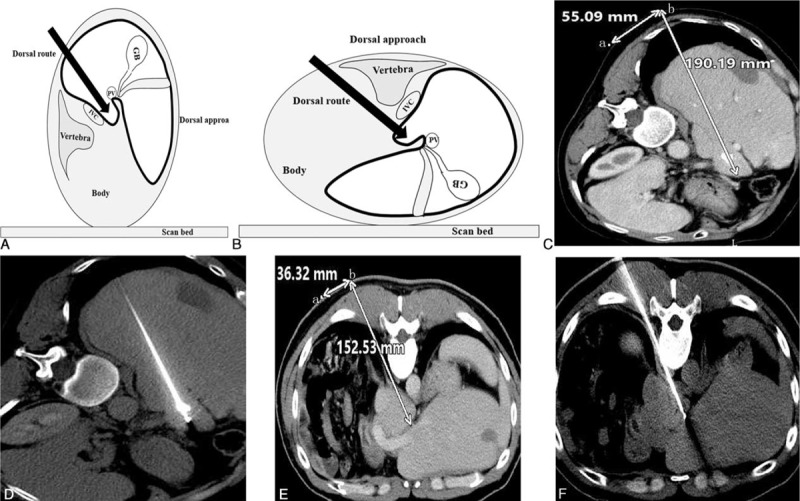
Illustration of dorsal approach. Figure A and B showed the posture for patients on the CT scan bed and the planned dorsal approach to the lesion. The puncture route was carefully designed on the preoperative contra-enhanced CT image (white arrow in liver in C and E). The appropriate angle and the depth of RFA probe (152.3 mm in C, 190.19 mm in E) were selected to avoid puncture injury to the vessels. The puncture site (b) was marked on skin (55.09 mm) with the help of an imaging marker (a). The probe was accurately positioned following the planned route (D and F). CT = computerized tomography, RFA = radiofrequency ablation.

The dorsal approach was used in cases that were not suitable for the right approach, via a dorsal puncture in the back with the patient in a prone position. The RFA probe passed through the erector spinae, phrenic feet, and retroperitoneal space into the caudate lobe tumor. Care was taken to avoid puncturing the inferior vena cava during the dorsal approach and respiratory depression for the limitation of movement of thorax (Fig. 3).

### RFA Procedure

**FIGURE 4:**
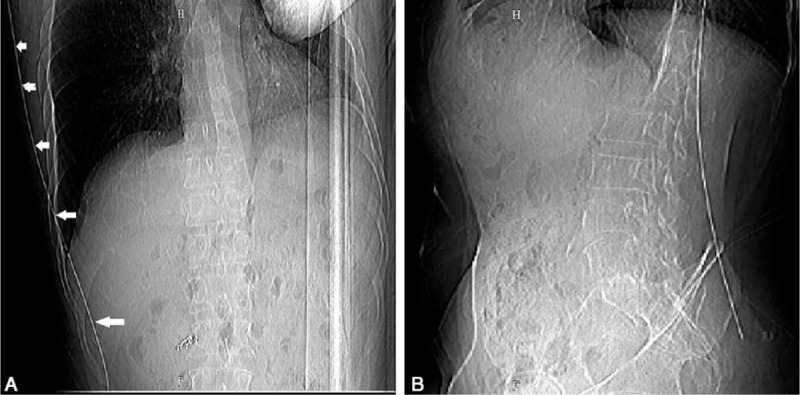
Imaging marker on the CT image. A single slim wire was positioned on the surface as the imaging marker; the wire (white arrows, A and B) was visible on CT images. It was used for locating the puncture. CT = computerized tomography.

The entire procedure was performed under intravenous anesthesia that was monitored by anesthetists. Patients were placed in a prone or left lateral position on the CT bed. Based on the preoperative CT or MRI, the operator was aware of the location of the tumor lesions and positioned a single slim wire (length 30 cm, diameter 0.12 cm) on the surface of the skin along the body as the imaging marker (Fig. 4). Preoperative contrast-enhanced CT scanning using a 16-slice PHILIPS Brilliance TM Big Bore CT scanner (Koninklijke Philips N.V., Amsterdam, Netherlands) was used to clearly visualize the structure of the involved area, including the tumor lesions and vascular structure. Sequential scanning was performed from maximal inspiration of the diaphragm to the lower edge of the liver using a 5 mm slice gap. With the help of the slim wire and positioning on the CT bed, the operator designed the approach of the monopolar RFA probe (17-15s30F, STARmed Co, Ltd, GYEONGGL-DO, Korea).

**FIGURE 5:**
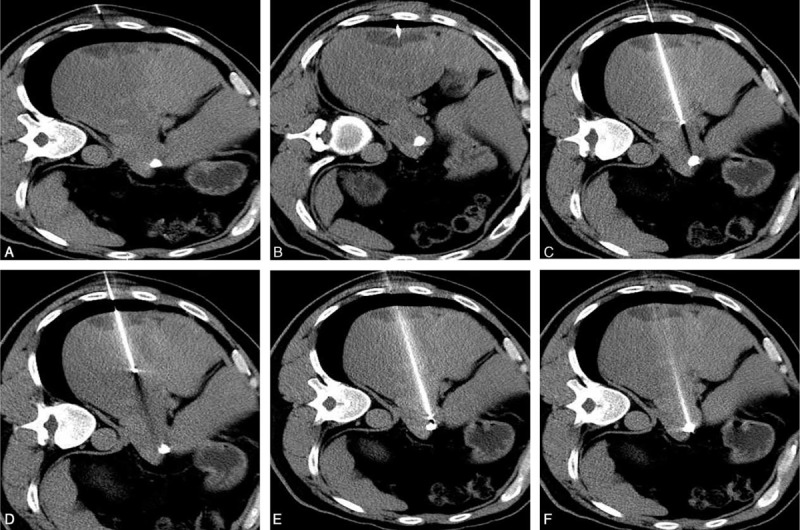
A series of CT scanning during the step-by-step puncture procedure. Three to six cycles of plain CT scanning would be performed during the step-by-step puncture procedure (A–F) to identify the position of RFA probe in the liver, until the probe was positioned accurately within the lesion. CT = computerized tomography, RFA = radiofrequency ablation.

After disinfection of the planned puncture site, a 17 G probe (the length of the exposed tip was determined by the lesion size) was inserted step-by-step from the puncture point along the planned approach (Fig. 5). Plain scanning was performed during the step-by-step procedure to confirm that the probe was on the correct track. If the probe was off-course, adjustments were made to correct the path toward the targeted lesion. Ablation time was mainly determined by the size of the tumor.

When the scheduled ablation time was completed, plain CT scanning was performed to evaluate the ablated zone. If necessary, contrast-enhanced CT scanning was also performed to map the residual activity of the lesion(s) and ensure ablation of a >5 mm margin surrounding the tumor. At the end of ablation, coagulation of the needle tract was performed to prevent metastasis along the tract. CT scanning was repeated to confirm that there was no bleeding in the liver capsule or abdomen after pulling the probe out. The vital signs of the patient were closely monitored during ablation. If the patient suffered postoperative pain, antalgic was administered. Blood loss in the procedure was estimated by the size of bloodstain in sterile gauze.

### Follow-Up

Planned patient follow-up was performed before the operation, 3 weeks after surgery and then every 3 months postsurgery. Patient follow-up included a contrast-enhanced CT examination of the abdomen, clinical examination, and blood samples. Residual tumors presented enhanced regions within the tumor on contrast-enhanced CT or MRI. Local recurrence was considered when new lesions were observed adjacent to the ablated area. MRI was carried out if contrast-enhanced CT could not clearly indicate the presence or absence of a residual tumor or metastases. After 3 weeks, the ablated area would be clearly mapped on the contrast-enhanced CT or MR images. If there was residual tumor, another ablation would be performed. Accordingly, another follow-up after 3 weeks would also be performed. If there was no residual tumor, the next follow-up was performed in 3 months after ablation. Every 3 months follow-up was to map the recurrence. Additional RFA treatment was provided to patients with lesions in other sites of liver. Major complications were defined as complications, which prolonged hospitalization, led to unplanned increases in the level of medical care, admission to hospital for therapy, and permanent adverse sequelae or death. Classification for severity of complications referred to WHO toxicity grading criteria.^8^

## RESULTS

### Clinical Characteristics

The 7 patients who underwent RFA ranged in age from 29 to 69 years (median, 52 years). Two patients with hepatocellular carcinoma (HCC) had previously undergone surgical resection and TACE. One patient with HCC had undergone TACE and surgical resection before RFA. Two patients with lung cancer had accepted chemotherapy and surgical resection of the primary tumor. One patient with HCC and another with liver metastases (adenocarcinoma) of unknown primary origin directly underwent RFA. A total of 8 hepatic caudate lobe lesions were treated in the 7 patients, using RFA in 6 cases and RFA and PEI in 1 case. Median tumor diameter was 29 mm (range, 18–69 mm). A right approach was used in 7 cases and a dorsal approach in 1 case; further details were provided in Table 2.

**TABLE 2:**
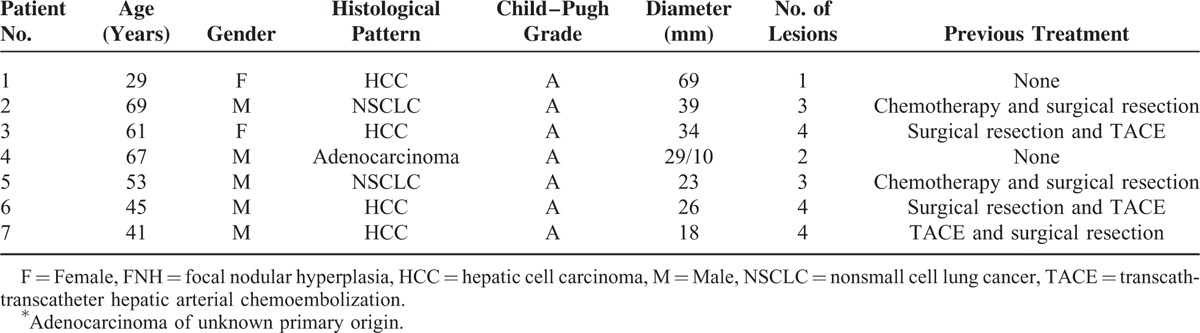
Clinicopathological Features of the 7 Patients With Caudate Lobe Tumors Who Underwent Radiofrequency Ablation

### Treatment Effectiveness

**FIGURE 6:**
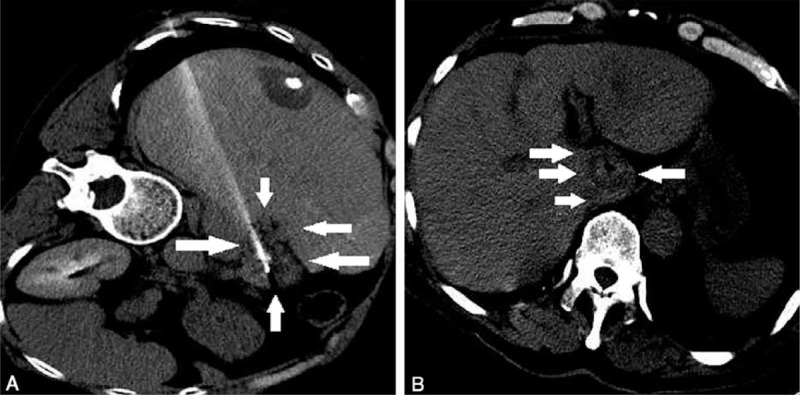
CT images after ablation. Plain CT scanning was performed after ablation to mark residual tumor. The boundary of ablated area (white arrows) was clearly visible on CT images (A and B). If the distance to the lesion was <5 mm, additional ablation would be considered. CT = computerized tomography.

**FIGURE 7:**
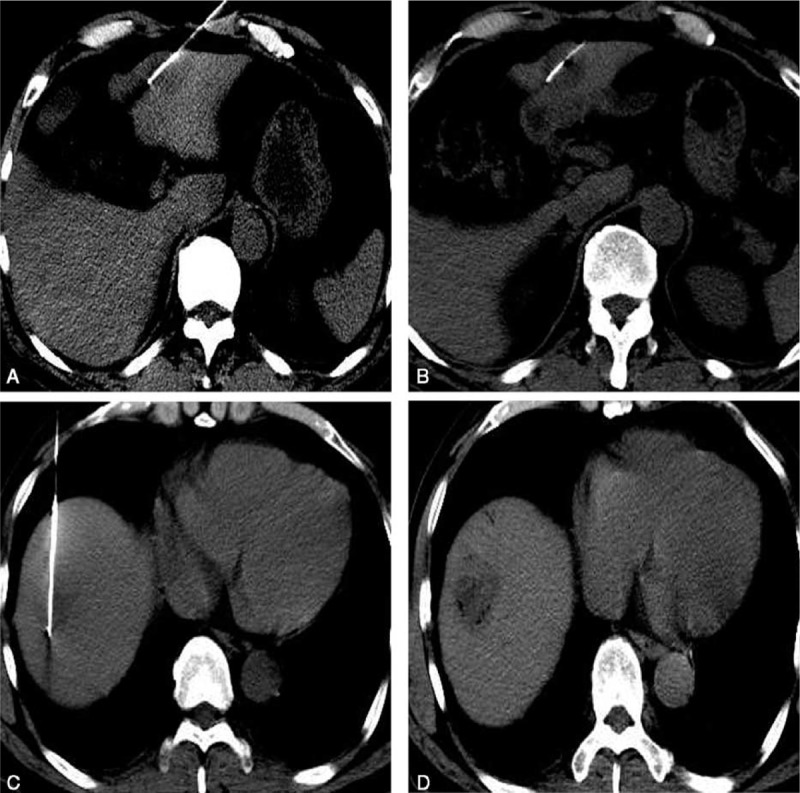
Additional ablation of other lesions in the liver. RFA ablation was performed on lesions in S3 and S8 (A, C). Tumors were completely inactivated after ablation (B, D). RFA = radiofrequency ablation.

All ablations were performed under the same CT guidance by the same radiologist with 14 years experience in radiology and operator with 9 years experience in tumor ablation therapy. The median duration of the procedure was 64 min (range, 59–102 min). Median blood loss was 10 mL (range, 8–16 mL), mainly as a result of puncture injury. All lesions were completely ablated (8/8, 100%) based on CT scanning immediately after RFA (Fig. 6). For patients with multiple lesions in other sites of liver, RFA was subsequently performed on the other lesions (Fig. 7). The median hospitalization time was 4 days (range, 2–5 days).

Median follow-up time was 8 months (range, 3–11 months). During the follow-up period, no recurrence was observed at the site of RFA, as shown in Table 3. Tumors developed at previous uninvolved regions of the liver in 6 patients (86%), extrahepatic tumor recurrence was diagnosed in 4 patients (57%), and tumor progression occurred in 5 patients (71%). All patients completed follow-up and were still alive by the date of last follow-up (November 26, 2013).

**TABLE 3:**
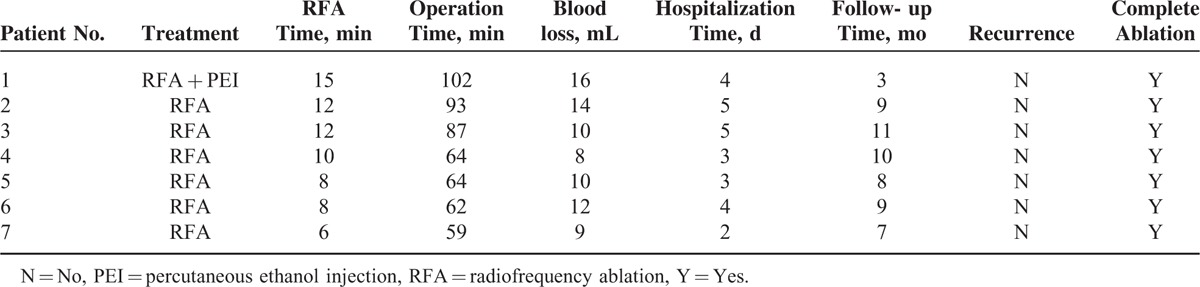
Effectiveness of RFA Treatment in the 7 Patients With Caudate Lobe Tumors

### Complications

There were no major complications, grade 3 to 4 toxicities in the liver, heart, or kidney, or procedure-related deaths after RFA. The most frequent minor complications reported included fever, mild-to-moderate right upper quadrant pain, fatigue, nausea, and vomiting. The overall incidence of treatment-related adverse effects was 7/7 (100%). Of the 7 patients, 7 (100%) developed grade 1 fever and chills 2 to 3 days after RFA; 3 patients (43%) developed grade I right upper quadrant pain, 3 patients (43%) developed grade II right upper quadrant pain, and 1 patient (14%) developed grade III right upper quadrant pain; 3 patients (43%) developed fatigue; 4 patients (57%) developed grade I nausea and vomiting after treatment and 3 patients (43%) developed grade II nausea. These reactions were easily controlled with symptomatic therapies and usually subsided within 1 to 4 days.

## DISCUSSION

Due to the deep location and position close to complex regional structures, it is highly challenging to resect tumors in the caudate lobe.^3,9^ Since the emergence of the RFA technique, increasing numbers of clinical practice guidelines have recommended this method as a radical treatment modality for hepatic carcinoma.^7^ Laparoscopic RFA, intraoperative RFA, or percutaneous RFA under ultrasound guidance are the most frequently applied RFA techniques.^6,10,11^ However, the rate of recurrence for caudate lobe tumors after laparoscopic or open RFA under ultrasound guidance is higher than that of surgical resection.^11,12^ The reason for it is that the vapor produced in tumor ablation bedims the vision and the boundary of ablated zone under ultrasound guidance. But CT imaging could resolve it well and clearly map the ablated area after RFA with 3-dimensional vision. Therefore, we performed this exploratory study to show a novel modality, CT-guided percutaneous step-by-step RFA, for the treatment of tumors in the caudate lobe.

To ensure safety, careful patient selection on the basis of CT or MR imaging is important. In the present study, the cohort included patients with primary liver cancer and patients with liver metastases, who had solitary or multiple lesions that were not suitable for surgical resection or who had refused surgery. However, these patients were suitable candidates for RFA treatment. Four patients had HCC, 2 patients had metastases to the liver from primary lung cancer and another patient had liver metastases (adenocarcinoma) of unknown primary origin. Of these 7 patients, 5 patients had undergone previous treatments, whereas the other 2 had not. The median tumor diameter was 29 mm; therefore, RFA could completely ablate the lesions. Only 1 lesion was 69 mm and received additional PEI to RFA. Multiple lesions were not a contraindication for RFA and also required a step-by-step RFA technique. If necessary, additional RFA was performed on residual lesions.

It is also essential to select an appropriate posture and puncture site to avoid vessel or bile duct injury. We commonly selected a prone or left lateral position and right or dorsal approach. An anterior approach under CT guidance through the left lobe of the liver has a high risk of lethal puncture injury to blood vessels or bile ducts. Contrast-enhanced CT provides clear images of the vessels on the puncture track and around the lesions in the caudate lobe. We created a planned approach for puncture on the CT images with the help of the PHILIPS Brilliance CT Imaging system. A dorsal approach was mainly applied in patients with a “fat” caudate lobe as a result of previous surgical resection or in whom vessels obstructed a right approach. However, we mainly selected the right approach, which directly entered the liver rather than the abdomen and therefore reduced the risk of puncture injury and associated complications. Once the planned puncture approach was selected, the most important issue was to avoid vessel injury during the procedure. Three to six cycles of plain CT scanning were strongly recommended during each step of insertion to identify the position of the RFA probe in the liver. Additionally, we aimed to insert the probe into the center of the lesion to achieve complete ablation, and the tip of the needle should pass through the distant boundary of the lesion to achieve an at least 5 mm safe margin.

Resection of caudate lobe tumors remains highly challenging to perform, requiring a long surgical time, and leading to high blood loss. Ishiko et al reported that the mean operative time for RFA using hand-assisted laparoscopic surgery for the treatment of HCC in the caudate lobe was 232 ± 68 min with a mean intraoperative blood loss of <89 ± 143 mL.^10^ Tanaka et al reported a higher level of intraoperative blood loss (2332.5 ± 1695.5 mL) and a longer operation time (399.5 ± 123.5 min) for surgical resection of caudate lobe tumors.^13^ In recent years, Yang et al reported a median operative time of 230 ± 43.6 min (range, 170–300 min) and median intraoperative blood loss of 606.6 ± 266.3 mL (range, 350–1200 mL) in surgery.^4^ However, due to the advantages of fast CT plain scanning, the procedure time for RFA was lower in this study, which also greatly decreased blood loss during the procedure. In the present study, the median operative time was 64 min (range, 59–102 min) and median blood loss, mainly as a result of the puncture injury, was 10 mL (range, 8–16 mL). Yang et al reported the median length of postoperative hospital stay was 12.6 ± 2.9 days for patients undergoing hand-assisted laparoscopic surgery, whereas only 4 days (range, 2–5 days) for patients undergoing RFA in this study.

Complications and recurrence rates are important factors impacting the survival of patients with caudate lobe tumors. In a cohort of 5 patients undergoing RFA with hand-assisted laparoscopic surgery, Ishiko et al reported that 2 patients developed subcutaneous hematoma and 1 patient suffered minor bile leakage.^10^ Peng et al performed percutaneous ultrasound-guided RFA on caudate lobe tumors and all 17 patients developed moderate fever and 8 patients developed moderate abdominal pain; however, the rate of recurrence was only 14%.^11^ Kariyama et al also performed ultrasound-guided percutaneous RFA on 50 consecutive patients with HCC located in the caudate lobe; no mortality or major complications were observed after RFA and the rate of local recurrence rate was 12%.^6^ In the present study, fever, pain, fatigue, nausea, and vomiting were common complications, but not serious. Tumor necrosis after ablation and intravenous anesthesia were responsible for that but symptomatic treatment would resolve them. Local recurrence mainly occurs when the ablation boundary cannot be confirmed by ultrasound or plain CT scan or a heat-sink effect in relation to the proximity of the tumor to major vessels. To avoid incomplete ablation, contrast-enhanced CT scanning after ablation to identify residual lesions would resolve the problem. In the present study, recurrence was not observed in any patient during the median 8 months follow-up period (range 3–11 months).

In conclusion, percutaneous step-by-step RFA under CT guidance is a novel and effective minimally invasive therapy for patients with tumors in the hepatic caudate lobe. Well-designed puncture route, appropriate posture, careful step-by-step insertion, and CT validation ensure the safety of the procedure.

## References

[1] Kim HO Kim SK Son BH Intraoperative radiofrequency ablation with or without tumorectomy for hepatocellular carcinoma in locations difficult for a percutaneous approach. *Hepatobiliary Pancreat Dis Int* 2009; 8:591–596. 20007075

[2] Abdalla EK Vauthey JN Couinaud C The caudate lobe of the liver: implications of embryology and anatomy for surgery. *Surg Oncol Clin N Am* 2002; 11:835–848. 12607574 10.1016/s1055-3207(02)00035-2

[3] Chaib E Ribeiro MA Jr Silva Fde S Surgical approach for hepatic caudate lobectomy: review of 401 cases. *J Am Coll Surg* 2007; 204:118–127. 17189120 10.1016/j.jamcollsurg.2006.09.020

[4] Yang JH Gu J Dong P Isolated complete caudate lobectomy for hepatic tumor of the anterior transhepatic approach: surgical approaches and perioperative outcomes. *World J Surg Oncol* 2013; 11:197. 23947911 10.1186/1477-7819-11-197 PMC3765966

[5] Yamamoto T Kubo S Shuto T Surgical strategy for hepatocellular carcinoma originating in the caudate lobe. *Surgery* 2004; 135:595–603. 15179365 10.1016/j.surg.2003.10.015

[6] Kariyama K Nouso K Wakuta A Percutaneous radiofrequency ablation for treatment of hepatocellular carcinoma in the caudate lobe. *Am J Roentgenol* 2011; 197:W571–W575. 21940528 10.2214/AJR.11.6893

[7] Minami Y Kudo M Radiofrequency ablation of hepatocellular carcinoma: current status. *World J Radiol* 2010; 2:417–424. 21179308 10.4329/wjr.v2.i11.417 PMC3006479

[8] World Health Organization. WHO Handbook for Reporting Results of Cancer treatment. 1979; Geneva, Switzerland: World Health Organization, 15–22.

[9] Sakoda M Ueno S Kubo F Surgery for hepatocellular carcinoma located in the caudate lobe. *World J Surg* 2009; 33:1922–1926. 19582505 10.1007/s00268-009-0110-7

[10] Ishiko T Beppu T Sugiyama S Radiofrequency ablation with hand-assisted laparoscopic surgery for the treatment of hepatocellular carcinoma in the caudate lobe. *Surg Laparosc Endosc Percutaneous Tech* 2008; 18:272–276. 10.1097/SLE.0b013e31816a24bf 18574414

[11] Peng ZW Liang HH Chen MS Percutaneous radiofrequency ablation for the treatment of hepatocellular carcinoma in the caudate lobe. *Eur J Surg Oncol* 2008; 34:166–172. 17851020 10.1016/j.ejso.2007.08.004

[12] Thanos L Mylona S Galani P Overcoming the heat-sink phenomenon: successful radiofrequency thermal ablation of liver tumors in contact with blood vessels. *Diagn Intervent Radiol* 2008; 14:51–56. 18306146

[13] Tanaka S Shimada M Shirabe K Surgical outcome of patients with hepatocellular carcinoma originating in the caudate lobe. *Am J Surg* 2005; 190:451–455. 16105535 10.1016/j.amjsurg.2004.12.005

